# A cross-sectional study on schistosomiasis and soil-transmitted helminths in Mbita district, western Kenya using different copromicroscopic techniques

**DOI:** 10.1186/s13071-016-1368-x

**Published:** 2016-02-16

**Authors:** Annette I. Ng’etich, Fredrick O. Rawago, Walter G. Z. O. Jura, Pauline N. Mwinzi, Kimberly Y. Won, Maurice R. Odiere

**Affiliations:** Neglected Tropical Diseases Branch, Centre for Global Health Research, Kenya Medical Research Institute, P. O. Box 1578-40100, Kisumu, Kenya; Department of Zoology, Maseno University, PRIVATE BAG, Maseno, Kenya; Division of Parasitic Diseases and Malaria, Centers for Disease Control and Prevention, Atlanta, GA USA

**Keywords:** Diagnosis, Kato-Katz, Mini-Parasep, Modified Mini-FLOTAC, Sensitivity, Schistosomiasis

## Abstract

**Background:**

Identification of populations to be targeted for individual treatment and broad-spectrum therapy in schistosomiasis-endemic areas, assessment of therapy efficacy, morbidity, and evaluation of control strategies need to be based on reliable diagnostic tools. Kato-Katz is routinely used and remains the standard diagnostic technique for schistosomiasis, despite its many challenges. This study was conducted in Nyamanga village, Mbita, western Kenya, and evaluated the diagnostic performance of Kato-Katz, Mini-Parasep and modified Mini-FLOTAC techniques in detection of *Schistosoma mansoni* and soil-transmitted helminths (*Ascaris lumbricoides*, *Trichuris trichiura* and hookworm) ova.

**Methods:**

Stool samples from 132 individuals were screened for eggs of *S. mansoni* by the 3 techniques. Mini-Parasep® faecal parasite concentrator (Apacor Ltd, England), a single-use diagnostic device with a built-in filter for faecal concentration of helminth eggs by sedimentation was employed on stool samples fixed in 10 % formalin. A modified Mini-FLOTAC (University of Naples, Italy) was based on floatation of helminths eggs with two different solutions (FS2 and FS7) using a closed system (Fill-FLOTAC) with 5 % formalin. Kato-Katz was performed following WHO recommendation. Prevalence of *S. mansoni* and STH, sensitivity and degree of agreement among the 3 techniques were determined.

**Results:**

Prevalence of *S. mansoni* was 47.0 %, 34.1 % and 20.5 % by Mini-Parasep, Kato-Katz and modified Mini-FLOTAC FS7 techniques, respectively. Prevalence of any STH infection was 6.1 %, 3.0 %, 6.1 % and 6.8 % by Mini-Parasep, Kato-Katz, modified Mini-FLOTAC FS2 and modified Mini-FLOTAC FS7 techniques, respectively. Considering the pooled results of the three methods (Mini-Parasep, Kato-Katz and modified Mini-FLOTAC FS7) as diagnostic ‘gold’ standard, the sensitivity of Mini-Parasep, Kato-Katz and modified Mini-FLOTAC FS7 for *S. mansoni* was 77.5 %, 56.1 %, and 33.8 %, respectively. Mini-Parasep and modified Mini-FLOTAC FS7 techniques had moderate (κ = 0.46) and fairly good (κ = 0.25) agreements with Kato-Katz for *S. mansoni*, respectively. Mini-Parasep detected a higher proportion of light intensity *S. mansoni* infections compared to Kato-Katz, which detected high proportions of heavy infections. Mini-Parasep detected a similar mean number of *S. mansoni* eggs per gram (EPG) of stool compared to the standard Kato-Katz (62.9 vs 97.3; t _(131)_ = -0.49, *P* = 0.6265) and significantly higher EPG compared to the modified Mini-FLOTAC FS7 (62.9 vs 34.6; t _(131)_ = 5.39, *P* < 0.0001).

**Conclusions:**

The high sensitivity of Mini-Parasep suggests its promising potential as an alternative tool in enhancing diagnosis and in monitoring schistosomiasis transmission and determining endpoint of intervention programs, especially in low endemicity areas. Mini-Parasep is also easy to operate, safe and also permits work with fresh stool.

## Background

Schistosomiasis is a major parasitic disease associated with considerable morbidity in the developing world and may lead to sequelae of acute and chronic infection. An estimated 207 million people in 74 countries are infected with schistosomiasis [1], with the majority, 90 % of the global cases residing in sub-Saharan Africa [2, 3]. It is estimated that more than 9.1 million Kenyans are at risk of infection with schistosomiasis [4] with highest infection rates found among adolescents aged 10–19 years, though high rates of infection have also been established among adults in rural areas who are employed in occupations associated with water contact [5].

Identification of populations to be targeted for individual treatment and broad-spectrum therapy in schistosomiasis-endemic areas, assessment of therapy efficacy, morbidity, and evaluation of control strategies need to be based on reliable and available diagnostic tools [6]. The gold standard for diagnosis of such a helminth infection is detection of characteristic parasite eggs in stool. Faecal egg count (FEC) techniques are widely used for parasitological diagnosis in humans and animals to assess the number of parasitic elements (eggs, larvae, oocysts) per gram of faeces (EPG/LPG/OPG) [7]. The Kato-Katz technique, despite its known limitations, is routinely used and remains the standard diagnostic technique for schistosomiasis. Kato-Katz faces several challenges, among them, patient compliance especially when more than one stool sample is required on consecutive days, and insufficient sensitivity especially when helminth infection intensities are low and eggs are highly clustered [8, 9]. In light of these challenges, use of Kato-Katz as a confirmatory test for diagnosis of intestinal helminths may contribute to significant increase in misdiagnosis of intestinal helminth infections and could lead further to misestimation of the prevalence of infection. Such diagnosis outcomes may negatively influence important mass drug administration (MDA) decisions. Nevertheless, the newly introduced Mini-FLOTAC (Prof. Giuseppe Cringoli, University of Naples Federico II, Naples, Italy) and Mini-Parasep (Apacor Ltd, Berkshire, England) may overcome this constraint. Studies using the Mini-FLOTAC have shown a higher sensitivity in the diagnosis of human hookworm infections which is generally underestimated when using Kato-Katz thick smears due to the rapid disintegration of hookworm eggs and the constraint to read slides within 30 min after preparation [10, 11]. Further, the Mini-FLOTAC does not require any expensive equipment or energy source, and so can be comfortably used in developing countries for larger studies [10]. The use of Mini-Parasep in epidemiological studies and control of intestinal parasitic infections has shown promising results [12, 13].

Efforts to identify an ‘ideal’ or alternative diagnostic technique based on faecal egg detection need to be strengthened, considering the many MDA programs currently being carried out. Successful treatment programs are likely to create a shift from heavy to light intensity helminth infections making it increasingly difficult to detect eggs in stool samples. The main objective of this study was to compare the performance of the Kato-Katz, Mini-Parasep and modified Mini-FLOTAC techniques for the accurate detection of *S. mansoni* and STH infections.

## Methods

### Study site

This study was conducted in Nyamanga, a beach village in Mbita District which borders Lake Victoria, between the months of May and June 2014. Mbita District has a population estimated at 111,409 [14]. Rainfall pattern in Mbita is seasonally bimodal, with the heaviest rains falling from March through May, and the shorter rainy season occurring between September and December. Previous studies in Mbita have shown high prevalence of *S. mansoni* among school children and community members residing close to Lake Victoria [15–17]. Occupational hazards associated with the lake such as sand harvesting, fishing and car washing and use of lake water for domestic purposes predispose individuals living close to the lake shores to schistosomiasis.

### Study design

This was a cross-sectional study nested within a larger ongoing project designed to evaluate the impact of integrated control programs for schistosomiasis and soil-transmitted helminths in Mbita district. Baseline prevalence and intensity (mean number of eggs per gram of faeces, EPG ± SE) of *S. mansoni* infection in the study village (located within 5 km from the lakeshore) were 36.4 % and 54.5 ± 12.2, respectively. Community health workers (CHWs) together with project staff mobilised individuals to enroll in the study.

### Parasitological assessment

A single stool sample was obtained from each participant (n = 132). Each stool was thoroughly mixed in a specimen cup using a wooden spatula and 0.5 g of the sample weighed using a digital weighing scale (CS 200, Ohaus Corporation USA) and transferred to the flat-bottom tube of the Mini-Parasep kit. 2.5 mL of 10 % formalin was then added to the weighed stool, mixed using a wooden spatula, and the Mini-Parasep kit was tightly closed. For the modified Mini-FLOTAC technique, one gram of the same stool sample was weighed and placed in a 15 mL centrifuge tube, and 4 mL of 5 % formalin was added. The stool and formalin mixture was stirred using a wooden spatula, and then tightly closed. The remaining stool in the specimen cup was examined by the Kato-Katz method within 5 h of collection. All collected samples were packed in cooler boxes and transported to the Division of Vector-Borne and Neglected Tropical Diseases (DVBNTD) laboratory in Homa-bay for Kato-Katz microscopy or temporary storage (for Mini-Parasep and Mini-FLOTAC). Mini-Parasep and Mini-FLOTAC samples were processed one month after collection at the NTD Parasitology laboratory, KEMRI’s Centre for Global Health Research (CGHR), Kisumu. Quality control was performed by an independent microscopist on 10 % of randomly chosen slides.

### Kato-Katz technique

All stool samples were analyzed in duplicate by the Kato-Katz method for detection of eggs of *S. mansoni, A. lumbricoides, T. trichiura* and hookworms [18]. Approximately 41.7 mg of faeces was used for each slide. Slides were allowed to clear for 20 min prior to microscopic examination. Arithmetic means were calculated for the two slides and intensity of infection was expressed as EPG of faeces.

### Mini-Parasep technique

**Fig. 1:**
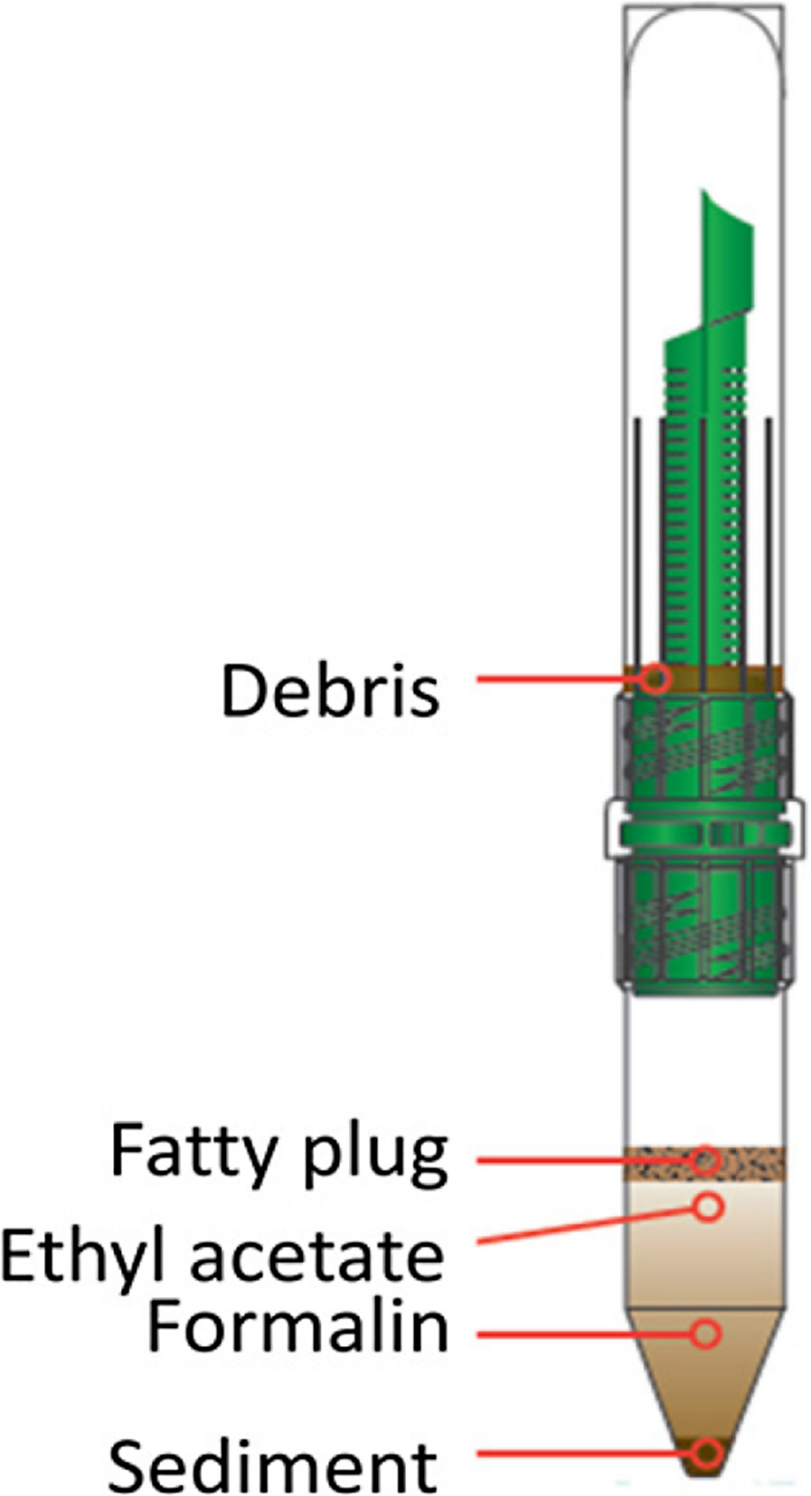
Mini-Parasep kit showing sediment, supernatant and debris after centrifugation (Apacor Ltd instruction sheet)

Mini-Parasep® faecal parasite concentrator (Apacor Ltd, England) is a single-use diagnostic device for faecal concentration of helminth eggs and larvae/protozoan cysts and oocysts by sedimentation (Fig. 1). It has a flat bottom tube allowing the mixing of stool with a solvent (ethyl acetate), a sedimentation cone, where deposition of helminth eggs and protozoan cysts occur after centrifugation, and a built-in filter for filtration. Briefly, 1 mL of ethyl acetate was added to the formalin + stool sample mixture in the Mini-Parasep tube, and shaken well. The Mini-Parasep kit was then inverted, and centrifuged at 1000 g for 4 min. After centrifugation, the fatty plug was removed, the supernatant decanted and the sediment was resuspended to 0.5 mL with 10 % formalin. 50 μL of the resuspended sediment was used to prepare duplicate microscope slides. The final EPG was obtained by multiplying the total number of eggs from the two slides by a factor 10.

### Mini-FLOTAC technique

**Fig. 2:**
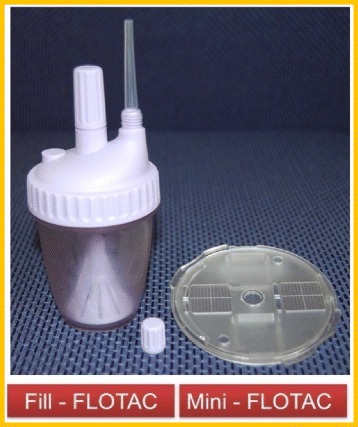
The Fill-FLOTAC and Mini-FLOTAC kit [22]

This technique utilises a new simplified device that has been developed to improve the quality of microscopic diagnosis of intestinal parasites (Fig. 2). It is based on floatation of helminth eggs using floatation solutions with different specific gravities (s.g). Saturated saline (sodium chloride, s.g 1.2) was used as floatation solution 2 (FS2), while saturated zinc sulphate, s.g 1.35 was used as floatation solution 7 (FS7). Mini-FLOTAC disc allows optimal examination of faecal suspensions while the fill-FLOTAC container permits performance of homogenization, filtration and filling processes.

### Modification of the Mini-FLOTAC technique

Our study employed a modified Mini-FLOTAC technique which involved 1 g of faeces that was collected and fixed with 4 ml of 5 % formalin. The standard protocol for the Mini-FLOTAC technique uses 2 g of the faecal samples that can be examined fresh or fixed with 5 % formalin (1 part of faeces + 1 part of 5 % formalin). The Mini-FLOTAC protocol does not involve centrifugation and provides the use of Fill-FLOTAC as it was created for basic laboratories with limited resources (i.e. where neither centrifugation nor other basic equipment are available). In our study, samples were stored for approximately 1 month and centrifuged before examination. The standard Mini-FLOTAC protocol recommends that samples be examined within 3 weeks of collection [19] with no additional centrifugation step recommended for sample processing. The modifications in our study were necessitated by the fact that we used preserved as opposed to fresh stool samples; the higher ratio of fixative to stool was considered necessary to prevent any hatching of eggs due to the slightly longer preservation time (1 month).

Since saturated saline (FS2) is recommended for diagnosis of STHs and saturated zinc sulphate for *S. mansoni* [20], the two solutions were tested in this setting where both STH and *S. mansoni* are endemic. Two Mini-FLOTAC procedures were therefore performed for each sample, one filled with faecal sediment in FS2 and the other filled with faecal sediment in FS7. Briefly, the preserved stool sample was centrifuged at 1000 g for 4 min and the supernatant discarded. 19 mL of the floatation solution, either saturated sodium chloride, specific gravity 1.2 (FS2) or saturated zinc sulphate, specific gravity 1.35 (FS7), together with the sediment were transferred to the fill-FLOTAC container, homogenised, and then loaded to the Mini-FLOTAC chambers. An average of 10 min was needed for the helminth eggs to float before reading and translating the reading disk. Eggs of intestinal helminths were identified and counted within the grids. Final EPG was obtained by multiplying the total number of eggs from chambers 1 and 2 of the Mini-FLOTAC disc by a factor 10.

### Ethics, consent and permissions

Informed consent (and assent where necessary) was obtained from study participants. All individuals infected with schistosomiasis were treated with 40 mg/Kg praziquantel (PZQ) while those infected with any STH were treated with 400 mg albendazole (ALB). Prior to the current study, there were two school-based national deworming exercises conducted in 2009 and 2013, where a single dose of ALB (400 mg) was administered. The current study was reviewed and approved by the Scientific and Ethical Review Committees (ERC) of the Kenya Medical Research Institute (KEMRI, SSC No. 2185), and reliance clearance from the Institutional Review Board of the US Centers for Disease Control and Prevention (CDC).

### Statistical analysis

All analyses were performed using SAS statistical software (v. 9.2; SAS Institute Inc., Cary, NC, USA) and P values < 0.05 were considered statistically significant. The sensitivity (proportion of true positives in the population) was calculated for each of the three methods, considering the combined results from the individual methods as the diagnostic ‘gold’ standard, an approach that maximised sensitivity values. Specificity was estimated at 100 % for each method. The prevalence of STH was too low for any meaningful sensitivity and specificity analyses. Cohen’s Kappa (k) statistic was employed to assess the degree of agreement among the three diagnostic techniques, with the strength of agreement determined using previously proposed criteria: ≤ 0 = poor, 0.01-0.20 = slight, 0.21-0.40 = fair, 0.41-0.60 = moderate, 0.61-0.80 = substantial and 0.81-1 = almost perfect [21]. Further, Spearman’s correlation coefficient was used to measure agreement among techniques based on egg counts. The mean EPG and standard error were calculated for the three different techniques. Since EPG values were overdispersed, the values were log-transformed (ln) for analyses. Differences between the mean EPG values (using the log-transformed data) between techniques were analyzed using paired samples T tests. Only non-transformed arithmetic means are reported. Intensity of helminth infection (egg density) was further categorised according to WHO-proposed thresholds for the classification of individuals with helminth infections [18].

## Results

One hundred and thirty-two individuals aged between 3 and 84 years (mean and median age was 23.5 and 14 years, respectively), were enrolled in the survey, of which 63 (47.7 %) were males.

### Prevalence and intensity of helminth infections

Results of the parasitological survey are shown in Table 1. Mini-Parasep detected a higher proportion of individuals positive for *S. mansoni* infection than Kato-Katz and Mini-FLOTAC FS7 techniques, whereas Mini-FLOTAC FS2 did not detect any *S. mansoni* ova (Table 1). Mini-FOTAC FS2, FS7 and Kato-Katz detected fairly equal proportions of STH infections (overlapping CIs) (Table 1).

**Table 1:** Prevalence and intensity of *Schistosoma mansoni* and soil-transmitted helminths by Mini-Parasep, Kato-Katz, Mini-FLOTAC FS2 and Mini-FLOTAC FS7 for stool samples collected in 2014, in Mbita, western Kenya (n = 132)^a^

Species	% Prevalence, (95 % CI)^b^	Intensity Threshold Prevalence, (%)				Egg density (EPG)^c^
		Uninfected	Light	Moderate	Heavy	
*Schistosoma mansoni*						
Mini-Parasep	46.9 (38.2-55.9)	53.0	38.6	7.6	0.8	62.9 ± 13.8
Kato-Katz	34.1 (26.1-42.8)	65.9	24.2	7.6	2.3	97.3 ± 18.5
Mini-FLOTAC (FS2)	0.0	0.0	0.0	0.0	0.0	0.0
Mini-FLOTAC (FS7)	20.5 (13.9-28.4)	79.6	18.9	1.5	0.0	34.6 ± 10.9
Any STH infection						
Mini-Parasep	6.1 (2.7-11.6)	93.9	6.1	0.0	0.0	ND^d^
Kato-Katz	3.0 (0.8-7.6)	97.0	3.0	0.0	0.0	ND
Mini-FLOTAC (FS2)	6.1 (2.7-11.6)	93.9	6.1	0.0	0.0	ND
Mini-FLOTAC (FS7)	6.8 (3.2-12.6)	93.2	6.8	0.0	0.0	ND

^a^Sample size: 3-5 years-11, 6-18 years-55, ≥ 15 years-65, unknown age-1

^b^95 % CI = 95 % confidence interval

^c^Eggs per gram expressed as arithmetic mean ± SE

^d^Not determined

**Fig. 3:**
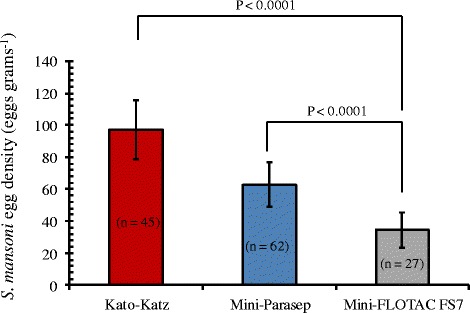
Comparison of egg densities for *Schistosoma mansoni* among Kato-Katz, Mini-Parasep and modified Mini-FLOTAC FS7 techniques. Values in parentheses represent the number positive for *S. mansoni* per technique

Kato-Katz detected more heavy intensity *S. mansoni* infections (≥400 EPG) compared to Mini-Parasep and modified Mini-FLOTAC FS7. Conversely, the Mini-Parasep technique detected a higher proportion of light intensity *S. mansoni* infections (1–99 EPG) relative to Kato-Katz and Mini-FLOTAC FS7 techniques (Table 1). Analyses of the log-transformed EPG data indicated differences in the mean *S. mansoni* egg densities among the techniques. The Mini-Parasep detected significantly higher mean number of eggs per gram of stool compared to the Mini-FLOTAC FS7 [t _(131)_ = 5.39, P < 0.0001], but similar to Kato-Katz [t _(131)_ = -0.49, P = 0.6265]; whereas Kato-Katz detected significantly higher *S. mansoni* EPG compared to the Mini-FLOTAC FS7 [t _(131)_ = 4.70, P < 0.0001] (Fig. 3).

#### Sensitivity of diagnostic tests

The Mini-Parasep technique detected *S. mansoni* ova with a higher sensitivity than the Kato-Katz and modified Mini-FLOTAC FS7 techniques. Considering the pooled results of the three methods as diagnostic ‘gold’ standard, Mini-Parasep had the highest sensitivity (77.5 %) for detecting *S. mansoni* eggs, whereas modified Mini-FLOTAC FS7 was the least sensitive (33.8 %) (Table 2).

**Table 2:** Sensitivity and negative predictive values (NPV) of the Mini-Parasep, Kato-Katz and Mini-FLOTAC FS7 techniques for the detection of *Schistosoma mansoni* in stool samples when considering the pooled results from the three methods as the diagnostic ‘gold’ standard (n = 132)^a^

	Sensitivity, % (95 % CI)	Specificity, %
Mini-Parasep	77.5 (66.8-86.1)	100^b^
Kato-Katz	56.1 (44.7-67.3)	100^b^
Mini-FLOTAC FS7	33.8 (23.6-45.2)	100^b^

^a^No *S. mansoni* eggs were detected by Mini-FLOTAC FS2

^b^We assumed 100 % specificity

#### Degree of agreement among techniques for detection of *S. mansoni*

A ‘moderate’ agreement was observed between Mini-Parasep and Kato-Katz for *S. mansoni* detection, and a ‘fair’ agreement between Mini FLOTAC FS7 and Kato-Katz (Table 3). The Mini-Parasep and Mini-FLOTAC FS7 recorded only a ‘slight’ agreement (Table 3). Analyses of data on the distribution of infection intensities between Kato-Katz and Mini-Parasep indicated that of the 51 *S. mansoni* infections classified as light by Mini-Parasep, 19 (37.3 %) of these were also classified as light by Kato-Katz, whereas 26 (50.9 %) and 6 (11.8 %) were classified as uninfected and moderate, respectively by Kato-Katz. Of the 70 *S. mansoni* samples that were classified as uninfected by Mini-Parasep, 61 (87.1 %) were also classified as uninfected by Kato-Katz, whereas 8 (11.5 %) and 1 (1.4 %) were classified as light and heavy, respectively by Kato-Katz. The one *S. mansoni* heavy infection by Mini-Parasep was classified as moderate by Kato-Katz. The 26 individuals who were identified as negative by the Kato-Katz method but positive by Mini-Parasep had a median egg count of 10 EPG (range = 5-85 EPG). The nine false-negative results from the Mini-Parasep technique had a median egg count of 24 EPG (range = 12-444 EPG) in the Kato-Katz smears. Of the three heavy *S. mansoni* infections by Kato-Katz, two (66.7 %) were classified as moderate and one (33.3 %) as uninfected by Mini-Parasep. On the other hand, 16 (59. 3 %) out of the 27 *S. mansoni* infections detected by modified Mini-FLOTAC FS7 were classified as positive infections by Mini-Parasep.

**Table 3:** Two-way contingency table showing the agreement between techniques for the detection of *Schistosoma mansoni* in stool samples from individuals from Mbita, western Kenya in 2014

	Positive	Negative	Total
Kato-Katz		Mini-Parasep	
Positive	36	9	45
Negative	26	61	87
Total	62	70	132
κ-agreement = 0.46 (*P* < 0.0001)			
Mini-FLOTAC FS7		Mini-Parasep	
Positive	16	11	27
Negative	46	59	105
Total	62	70	132
κ-agreement = 0.10 (*P* = 0.1953)			
Kato-Katz		Mini-FLOTAC FS7	
Positive	16	29	45
Negative	11	76	87
Total	27	105	132
κ-agreement = 0.25 (*P* = 0.0030)			

A similar pattern of agreement among techniques was revealed by correlation analyses on *S. mansoni* egg counts. The strongest correlation was observed between Mini-Parasep and Kato-Katz (*r*_*s*_ = 0.60, *n* = 132, *P* < 0.0001), followed by Kato-Katz and modified Mini-FLOTAC FS7 (*r*_*s*_ = 0.31, *n* = 132, *P* = 0.0003) and lastly by Mini-Parasep and modified Mini-FLOTAC FS7 (*r*_*s*_ = 0.25, *n* = 132, *P* = 0.0034).

## Discussion

This study highlights the potential of the recently developed Mini-Parasep and modified Mini-FLOTAC techniques in detecting helminth ova in stool samples relative to the routinely used WHO-recommended Kato-Katz technique. Findings from this study have shown a significant difference in performance among the three techniques in estimating prevalence for *S. mansoni*. Mini-Parasep performed better than Kato-Katz and Mini-FLOTAC in detecting *S. mansoni* infection. Worth noting is that the prevalence of any STH by the three techniques was fairly equal, though with a slightly lower value by the Kato-Katz technique. Of particular consideration is the higher sensitivity of the Mini-Parasep relative to the standard Kato-Katz technique in detecting *S. mansoni* infection. Mini-Parasep detected more light infections than Kato-Katz; while on the other hand, Kato-Katz detected more heavy infections than Mini-Parasep. There was a fair to moderate agreement between the Mini-FLOTAC FS7, Mini-Parasep and Kato-Katz techniques. Not only did the Mini-Parasep out-perform the Mini-FLOTAC FS7 and Kato-Katz in estimating the prevalence of *S. mansoni* and Kato-Katz, for any STHs, it also revealed similar *S. mansoni* egg density to the standard Kato-Katz and higher EPG than the Mini-FLOTAC FS7.

One plausible explanation for the superior performance of Mini-Parasep in detecting *S. mansoni* eggs could be the incorporation of ethyl acetate, known to remove fat and organic compounds resulting in more clearly examinable slides, hence increasing the chances of detecting eggs. On the other hand, *S. mansoni* eggs may disappear due to over-clearing by glycerin, especially when long delays occur between Kato-Katz thick smear preparation and microscopy. Furthermore, only a small amount of faecal material (41.7 mg) is processed for the Kato-Katz technique compared to the Mini-Parasep (0.5 g) techniques.

Studies using the FLOTAC and Mini-FLOTAC techniques in detecting *S. mansoni* and STHs have been largely successful with encouraging results [7, 10, 20, 22, 23]. In our study, the Mini-FLOTAC FS2 and FS7, and Kato-Katz performed equally (CIs overlapped for STH prevalence), even though FS2 is recommended for detection of STH. The equal performance by the Mini-FLOTAC FS2 and FS7 for STH and results for *S. mansoni* (higher EPG by Kato-Katz compared to FS7) is consistent with other studies [10, 22]. Our findings further support the observation that Mini-FLOTAC is a sensitive approach for helminth diagnosis [22], and its diagnostic sensitivity depends on the floatation solution used: FS2 for STHs whereas FS7 for *S. mansoni*. Diagnosis of intestinal helminth infections based on egg detection in stool using direct parasitological methods (such as Kato-Katz) can become unreliable among infected individuals who harbor few intestinal worms, since egg output is much lower than among heavily infected persons. Prior to our study, baseline *S. mansoni* prevalence in the study village was 36.4 %, categorizing it as a ‘moderate-risk’ community [24]. A single round of MDA with PZQ was conducted 5 months before our study and likely reduced the proportion of moderate and heavy intensity infections. Our results are consistent with the suggestion that the performance of Kato-Katz declines as prevalence and intensity declines and that faecal concentration methods may perform better in these situations. This indicates that in settings where deworming programs have been implemented and infection intensities have been reduced, diagnostic methods with high sensitivity are required.

The distribution of infection intensities for *S. mansoni* infections by Kato-Katz and Mini-Parasep in this study has several implications for monitoring and evaluating mass treatment programs. WHO recommends MDA with PZQ (for schistosomiasis) wherever the prevalence of infection exceeds 10 % [24]. Data from the current study shows that the Mini-Parasep technique performed better at detecting light *S. mansoni* infections (1-99 EPG) (38.6 % by Mini-Parasep vs 24.2 % by Kato-Katz); whereas Kato-Katz was better at detecting heavy *S. mansoni* infections (≥400 EPG) (2.3 % for Kato-Katz vs 0.8 % for Mini-Parasep). However, Mini-Parasep produced 9 false positive results with regard to *S. mansoni*-positive Kato-Katz thick smear readings. Important to note is that only one of these Kato-Katz readings had many eggs (nine eggs), the other eight had only 1–2 eggs, suggesting that mainly light *S. mansoni* infections were missed by Mini-Parasep. Nevertheless, our results suggest that although both Mini-Parasep and Kato-Katz missed light infections, fewer of these were missed by Mini-Parasep relative to Kato-Katz. In support of this, further analyses revealed that more than half of the 51 light infections (50.9 %) detected by Mini-Parasep were classified as uninfected by Kato-Katz, indicating that Kato-Katz would likely result in misdiagnosis of a majority of individuals with light infections. The implications of such misdiagnosis include misestimation of the prevalence of infection in a population, which would affect important MDA decisions, specifically the cut-offs prevalence thresholds and frequency of implementing MDA.

On the other hand, the WHO emphasises morbidity control in schistosomiasis. Existing evidence shows that the degree of morbidity is related to the intensity of infection, where individuals with heavy infection intensities tend to experience more severe health outcomes [25, 26]. Use of Mini-Parasep therefore may misclassify heavy infections. Caution may be required during interpretation of the impact of treatment, and during monitoring and evaluation of control programs. Additional studies are therefore needed to further validate the performance of Mini-Parasep in areas of high *S. mansoni* endemicity where heavy infections are likely to be common.

The Mini-FLOTAC FS7 detected significantly lower *S. mansoni* EPG than the Kato-Katz technique in our study (35 vs 97). A similar pattern was reported from Mwanza, Tanzania, where Mini-FLOTAC FS7 detected a slightly lower but not statistically different mean EPG for *S. mansoni* compared to the Kato-Katz (58 vs 71) [10]. It is noteworthy that although the Mini-FLOTAC technique has been used in several previous studies [10, 19, 22] with good results for the diagnosis of *S. mansoni*, it’s use for schistosomiasis diagnosis is not yet included in the list of parasites on the website (www.parassitologia.unina.it), because it’s validation requires additional studies. The poor performance of the modified Mini-FLOTAC FS7 for *S. mansoni* detection in our study seems to accord with this observation. On the other hand, the fact that the Mini-Parasep detected a similar number of *S. mansoni* eggs compared to Kato-Katz and higher than Mini-FLOTAC FS7 in the present study is encouraging indeed, considering that this is a relatively new technique. Intensity of infection remains an important aspect in helminth control as egg density is directly related to morbidity, and is an important indicator in monitoring the impact of control programs as well as drug efficacy [22, 27]. It is important to acknowledge that the recently developed point-of-care circulating cathodic antigen (POC-CCA) test also offers a viable alternative to Kato-Katz in moderate-to-heavy intensity infection areas [28]. However, whereas POC-CCA is specific in detecting schistosomiasis, Mini-Parasep has the added advantage of detecting STHs as well as other intestinal protozoa.

The performance of the two floatation solutions in detection of various helminth ova in our study is worth noting: whereas Mini-FLOTAC FS7 detected *S. mansoni*, Mini-FLOTAC FS2 did not. In addition, the Mini-FLOTAC FS2 detected more hookworm than the Zinc sulphate FS7 solution in our study (data not shown). This is consistent with other findings [10]. Differences in sensitivity of the solutions to helminth eggs are known to be associated with differences in the specific gravities of the solutions, which is crucial for floatation of the eggs [10]. The saturated saline (FS2) has been shown to perform better and is recommended for the diagnosis of STHs, while Zinc sulphate (FS7) is recommended for *S. mansoni* diagnosis [20].

## Conclusions

Our findings confirm that the prevalence of *S. mansoni* infections, and conceivably also STHs, may be underestimated by the Kato-Katz technique in ‘light-to-moderate risk’ communities, and suggests that the Mini-Parasep technique may improve the ability to diagnose intestinal schistosomiasis accurately in such settings. The high sensitivity of Mini-Parasep suggests its promise as an alternative diagnostic tool in enhancing diagnosis and in monitoring schistosomiasis transmission, and determining endpoint of intervention programs, especially in low endemicity areas. Such sensitive diagnostic techniques are needed more so now, considering the upscale of control interventions against neglected tropical diseases in accordance with the WHO goals set for the year 2020, which will likely reduce the prevalence and intensities of schistosomiasis and STH infections in endemic countries. Other additional advantages of the Mini-Parasep technique include the fact that it can be used for the simultaneous diagnosis of different helminths as well as intestinal protozoa. This is especially important considering that multiple-species parasitic infections are a common occurrence in the developing world. Mini-Parasep is also easy to operate, safe and permits work with fresh stool.

## Abbreviations

EPG: Eggs per gram of faeces
FS: Floatation solution
LPG: Larvae per gram of faeces
MDA: Mass drug administration
OPG: Oocysts per gram of faeces
POC-CCA: Point-of-care circulating cathodic antigen
SG: Specific gravity
STH: Soil transmitted helminths
